# Supplementary material for: MetaRibo-Seq measures translation in microbiomes
Source: Nat Commun. 2020 Jun 29;11:3268. doi: 10.1038/s41467-020-17081-z (PMC7324362; doi:10.1038/s41467-020-17081-z)
Supplement: Supplementary file 10 — Supplementary Data 7 [file 41467_2020_17081_MOESM10_ESM.zip › File2/Confidence_High_Taxonomy/39187_out.krona.html]

Javascript must be enabled to view this page.

members
magnitude
magnitudeUnassigned
count
unassigned
taxon
rank

39187\_out

35

35
2
superkingdom

1224
phylum
35

35
class
28216

order
80840
35

995019
family
35

40544
genus
35

40545

SRS048981\_contig\_number\_contig-100\_1900.45036SRS051031\_contig\_number\_contig-100\_6138.141291SRS053649\_contig\_number\_contig-100\_9171.105528SRS054905\_contig\_number\_23460SRS075021\_contig\_number\_4715SRS076804\_contig\_number\_4810SRS101376\_contig\_number\_28789SRS104036\_contig\_number\_contig-100\_1105.59605SRS104311\_contig\_number\_40137SRS104912\_contig\_number\_contig-100\_1221.117664SRS142923\_contig\_number\_23124SRS144506\_contig\_number\_48964SRS145497\_contig\_number\_contig-100\_39.99061SRS148424\_contig\_number\_28017
species
14

2

SRS045004\_contig\_number\_contig-100\_10488.126725SRS104327\_contig\_number\_4504
1897024
species

19
species
1574262

SRS019910\_contig\_number\_contig-100\_14439.100879SRS024075\_contig\_number\_5851SRS044535\_contig\_number\_16176SRS047044\_contig\_number\_contig-100\_10012.77869SRS049712\_contig\_number\_contig-100\_3686.128099SRS050026\_contig\_number\_contig-100\_1947.68605SRS050299\_contig\_number\_4793SRS054956\_contig\_number\_16054SRS055017\_contig\_number\_contig-100\_10384.146405SRS076756\_contig\_number\_19507SRS077086\_contig\_number\_contig-100\_1370.87051SRS078665\_contig\_number\_9579SRS098571\_contig\_number\_68084SRS105082\_contig\_number\_contig-100\_1193.122420SRS143876\_contig\_number\_39269SRS146764\_contig\_number\_32080SRS147271\_contig\_number\_contig-100\_8858.93911SRS147919\_contig\_number\_15165SRS148253\_contig\_number\_12426
